# P06-09 Data processing with the short questionnaire to assess health enhancing physical activity (SQUASH): an update

**DOI:** 10.1093/eurpub/ckac095.094

**Published:** 2022-08-29

**Authors:** Marjolein Duijvestijn, Ellen de Hollander, Saskia van den Berg, Wanda Wendel-Vos

**Affiliations:** Centre for Nutrition, Prevention and Health Services, National Institute for Public Health and the Environment, Bilthoven, The Netherlands

**Keywords:** SQUASH, physical activity, data processing

## Abstract

**Background:**

The Short Questionnaire to Assess Health Enhancing Physical Activity (SQUASH) is a widely used questionnaire, and used for monitoring prevalence rates of physical activity(PA) in the Netherlands. To provide a standardized protocol for data processing and analysis of the SQUASH, an analysis guide was published in 2004. However, since then, the compendium of Metabolic Equivalent (MET) values of PA has been updated, and new PA guidelines have been developed. The new PA guidelines differ from the old ones in terms of the appropriate amount of active time (150 minutes/week versus 5 days/week 30 minutes), decrease in cut-off point for moderate intensity (adults 18-54 years of age) and adding a bone- and muscle strengthening component. Therefore, the protocol for data processing and analysis of the SQUASH needs to be updated. In this study, results from the old and new protocol demonstrate the differences in adherence rates between the two sets of guidelines in the Netherlands for the adult population.

**Methods:**

Data of a nationally representative sample of 6942 participants aged 18 years and older were used to calculate adherence to the old and the new PA guidelines by using the original and the updated protocol. In the new protocol, the MET-values of the activities including sports were adjusted according to the 2011 Compendium. Moderate intense activity was defined as ≥ 3.0 MET irrespective of age and the bone and muscle strengthening component was added.

**Results:**

Adherence to the old Dutch PA guidelines is 48.1% among adults aged 18-54 years, and 74.4% among adults 55 years and older. For the new PA guidelines the adherence is 48.4% and 38.1% respectively. The large difference for adults 55 years and older is due to changes in the cut-off values for moderate-to-vigorous intensity PA and the addition of bone and muscle strengthening exercises.

**Conclusions:**

The updated protocol for data processing and analysis of the SQUASH describes the steps to calculate the new PA guidelines in a structured way and gives researchers the opportunity to work with the data from the SQUASH in a uniform way. The SPSS syntax for data processing is available at: www.sportenbewegenincijfers.nl/methoden.

